# Long-term preservation of pharyngeal swallowing function in MM2-cortical-type sporadic Creutzfeldt-Jakob disease

**DOI:** 10.1080/19336896.2021.1930851

**Published:** 2021-06-02

**Authors:** Yuichi Hayashi, Kenjiro Kunieda, Takuya Kudo, Akio Kimura, Ichiro Fujishima, Takayoshi Shimohata

**Affiliations:** a Department of Neurology, Gifu University Graduate School of Medicine, Gifu, Japan; b Department of Rehabilitation, Hamamatsu City Rehabilitation Hospital, Hamamatsu, Japan

**Keywords:** Creutzfeldt-Jakob disease, MM2-cortical-type, brainstem function, dysphagia, pseudobulbar palsy

## Abstract

Swallowing function in long-term survivors of Creutzfeldt-Jakob disease (CJD) has not been elucidated. Herein, we report a patient with MM2-cortical-type sporadic CJD (MM2C-type sCJD) with long-term preservation of pharyngeal swallowing function using videofluoroscopic (VF) examination of swallowing. A 55-year-old woman was admitted to hospital because of dyscalculia and memory disturbance 3 years after the onset of these symptoms. Neurological examination revealed dementia, extrapyramidal signs, and delusion. Diffusion-weighted MRI revealed bilateral hyperintensity in the basal ganglia and frontal, temporal, and parietal cortices. No mutation with the methionine homozygote at codon 129 was found on PRNP gene analysis. VF was performed 68 months after the onset. Although bolus transport from the oral cavity to the pharynx worsened, the pharyngeal swallowing function was preserved even 68 months after onset. Serial MRI examinations revealed no apparent atrophy of the brainstem. Single photon emission computed tomography revealed that the regional cerebral blood flow in the brainstem was preserved. These findings suggest that pseudobulbar palsy is the pathophysiology underlying dysphagia in long-term survivors of MM2C-type sCJD, probably owing to preserved brainstem function even in a state of akinetic mutism.

## Introduction

MM2-cortical-type sporadic Creutzfeldt-Jakob disease (MM2C-type sCJD) manifests as fatal dementia with a relatively slow progression. The frequency of MM2C-type sCJD is reportedly 2.0% and 6.7% in Caucasian and Japanese sCJD populations, respectively [1]. The average disease duration of MM2C-type sCJD is 15.7–20.6 months [1–3]; however, long-term survivors (more than 3 years) have also been reported [4,5]. The implementation of appropriate care for dysphagia is an important factor that enables long-term survival in patients with MM2C-type sCJD [5], and genetic CJD with V180I mutation in *PRNP* (V180I gCJD) [6].

Tube feeding, including gastrostomy, is particularly associated with long-term survival in Japanese patients with CJD [7]. On the other hand, our previous report showed that a patient with MM2C-type sCJD could orally ingest a fully supported diet without tube feeding for 46 months after the onset [5]. Swallowing function in long-term survivors of MM2C-type sCJD remains unknown. Additionally, few studies have reported on the optimal period for the continuation of oral intake in these patients.

New clinical diagnostic criteria were recently proposed for MM2C-type sCJD [8], as most of patients with MM2C-type sCJD do not meet the existing clinical criteria for the diagnosis of sCJD [9]. The clinical diagnosis of MM2C-type sCJD in surviving patients is based on the new diagnostic criteria [8]. Herein, we report the evaluation of swallowing function in a patient who was a long-term survivor of probable MM2C-type sCJD using videofluoroscopic (VF) examination of swallowing.

## Patient and methods

### Clinical summary

**Figure 1.**
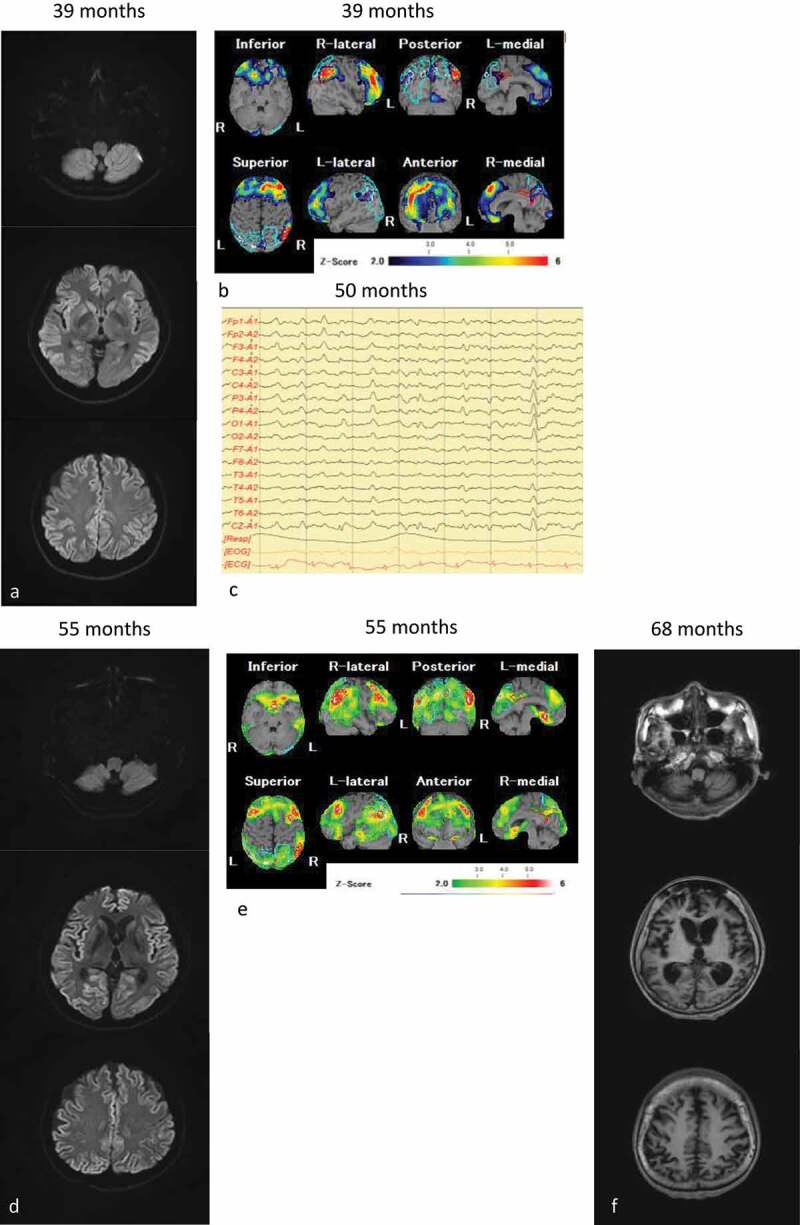
MRI, single photon emission computed tomography (SPECT) and electroencephalography of the patient. (a): Diffusion-weighted MRI (DW-MRI), and (b): SPECT images obtained 55 months after onset; (c): Electroencephalography obtained 50 months after onset; (d): DW-MRI, and E: SPECT images obtained 55 months after onset; (f): T1-weighted MRI obtained 68 months after onset. The eZIS analysis of SPECT images revealed decreased regional cerebral blood flow (rCBF). A higher Z-score indicated a lower rCBF. A Z-score of 2 to 6 is indicated by the green or black-to-red (lower rCBF) colour gradient. Panel A shows the bilateral hyperintense lesions in the frontal, temporal, and parietal cortices, and the basal ganglia. Panel B depicts the decreased rCBF in the frontal and parietal lobes. Panel C depicts the periodic sharp wave complexes. Panel D depicts bilateral hyperintensity in all cortices, and panel E shows preserved rCBF in the brainstem. Panel F depicts progressive cerebral atrophy, but no brainstem atrophy

A 55-year-old woman was admitted to the hospital 3 years after the onset of slowly progressive memory disturbance and dyscalculia in the absence of any relevant family history. She had hypothyroidism for 25 years, and her thyroid function was well controlled. Neurological examinations confirmed the diagnosis of dementia, with a Mini-Mental State Examination score of 22/30 points 38 months after the onset of symptoms, which deteriorated to 2/30 points 42 months after the onset of symptoms and revealed delusional jealousy and exaggerated tendon reflexes without the Babinski sign. The results of cerebrospinal fluid (CSF) examination were normal including the total tau levels and absence of 14-3-3 proteins. *PRNP* gene analysis revealed no mutation with the methionine homozygote at codon 129. Brain diffusion-weighted MR images (DW-MRI) showed bilateral hyperintense lesions in the frontal, temporal and parietal cortices, and basal ganglia (Figure 1(a)). Single photon emission computed tomography (SPECT) revealed decreased regional cerebral blood flow (rCBF) in the frontal and parietal lobes (Figure 1(b)). Cortical blindness was observed 48 months after onset. She was admitted to our hospital 49 months after onset. CSF examination revealed elevation in the total tau protein levels (1654 pg/mL). Prion proteins in the CSF were amplified using the real-time quaking-induced conversion methods [10]. The anti-N-terminal α-enolase antibody, which is associated with Hashimoto’s encephalitis [11], was no detected in the serum. Her symptoms did not improve following the administration of corticosteroid pulse therapy. Therefore, steroid-responsive encephalitis was excluded from the differential diagnosis. Periodic sharp wave complexes were observed on electroencephalography performed 50 months after onset (Figure 1(c)). DW-MRI revealed bilateral hyperintensity in all the cortices (Figure 1(d)), and SPECT showed preserved rCBF in the brainstem (Figure 1(e)). The patient’s clinical course and symptoms were consistent with probable MM2C-type sCJD [8]. Her condition reached the state of akinetic mutism 58 months after onset; however, she was fed orally by a caregiver.

### Evaluation of swallowing function

**Figure 2.**
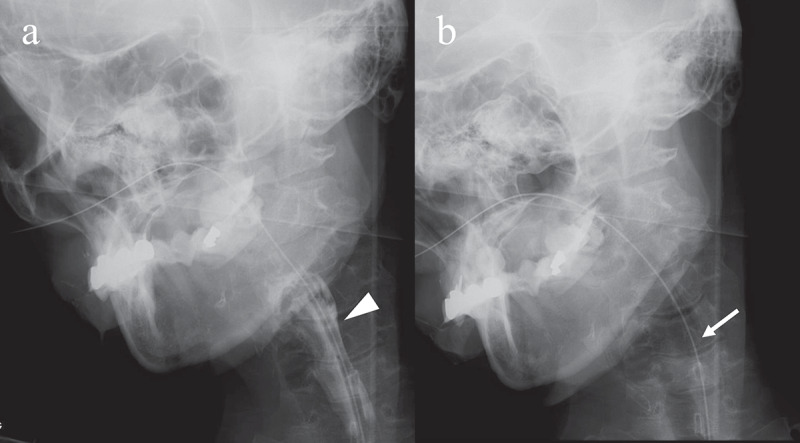
Videofluoroscopic examination of swallowing (VF) of the patient. VF of the patient in frontal view acquired 68 months after the onset of symptoms. (a) Once the swallowing reflex was triggered, the bolus passed through the pharynx into the upper oesophagus (arrowhead). (b) Pharyngeal residue or aspiration was not observed after swallowing (arrow)

The patient exhibited level 8 dysphagia (she could eat three meals by excluding food that is particularly difficult to swallow) based on the Food Intake LEVEL Scale (FILS) classification [12] 58 months after onset. She was hospitalized because of aspiration pneumonia at 68 months after onset. Her dysphagia was classified as level 3 on the FILS (swallowing training was performed using a small quantity of food) [12]. VF was performed after her pneumonia improved (Figure 2(a,b)). Although the bolus transport from the oral cavity to the pharynx had worsened, the pharyngeal swallowing function was preserved without pharyngeal residues or aspiration. At that time, brain MRI showed progressive cerebral atrophy, but brainstem atrophy was not apparent (Figure 1(f)). Although gastrostomy was performed, her family wanted her to continue ingesting a small amount of food orally with assistance. At that time, her ability to swallow was classified as level 4 [easy-to-swallow food less than the quantity of an orally ingested meal (enjoyment level)] on the FILS [12].

## Discussion

To the best our knowledge, this is the first report to evaluate swallowing function using VF in a long-term survivor of MM2C-type sCJD. The pharyngeal swallowing function, especially the brainstem swallowing reflex, may be preserved even in the akinetic state in some patients with MM2C-type sCJD.

According to our VF study, the swallowing pattern was indicative of pseudobulbar palsy. The oral stage of swallowing was impaired and bolus transport from the oral cavity to the pharynx was poor. Meanwhile, the pharyngeal swallowing function was preserved without pharyngeal residue and aspiration. The VF finding in the present patient is typical of pseudobulbar palsy due to bilateral cortico-bulbar tract impairment [13]. Serial SPECT studies did not reveal any apparent decrease rCBF in the pyramidal tract or the motor cortices; however, the bilateral cerebral cortices, including the motor cortices were atrophic on T1-weighted MRI performed 68 months after the onset of symptoms (Figure 1(f)).

Our report suggests that the preservation of brainstem function facilitates swallowing in patients with MM2C-type sCJD. The brainstem was preserved according to the serial MRI and SPECT studies, similar to that in patients with V180I gCJD [14]. Previous neuropathological studies in MM2C-type sCJD showed that brainstem was preserved [1,2], except of concomitance of the MM2-thalamic form (MM2C+T-type sCJD). The central pattern generators (CPGs) that control the pharyngeal phase of the swallowing sequence are located in the nucleus tractus solitarius [15]. The coordination of pharyngeal contraction and the opening of the upper oesophageal sphincter may also be preserved in cases without neurological impairment in brainstem function, akin to the current patient, because the CPGs were not impaired. A previous study showed that most patients with sCJD could not continue oral intake, and that received tube-feeding once they reached the akinetic state in Japan [7]. However, the brainstem is not usually affected as severely as the supra-tentorial structure in sCJD even on pathological examination [1,2]. We considered that the pharyngeal function may be preserved, although the oral stage of swallowing may be chiefly affected in patients with sCJD. Therefore, we emphasize that clinicians should consider evaluating the swallowing function with VF and/or videoendoscopic examination even if a patient with MM2C-type sCJD attains a state of akinetic mutism. Previous studies reported that the brainstem and cerebellum were preserved in MM2C-type sCJD [1,2] and that these findings supported the diagnosis of pseudobulbar palsy. Some patients with MM2C-type sCJD and V180I gCJD [16] could continue oral intake for a long time even in the akinetic state. The present patient’s family wished for her to continue the oral intake of nutrition. Continuing ingestion orally may be meaningful for families and caregivers based on accurate assessment of swallowing function. Appropriate care and swallowing techniques, such as oral care, ice massage, dietary modification, and postural adjustment, may be useful for avoiding adverse events such as aspiration pneumonia and continuing oral intake safely.

This study has some limitations. First, pathological analysis was not performed since the patient was alive. Therefore, further studies on the neuropathological findings of this patient are needed. Second, this was a single case report, necessitating future research and evaluation of additional cases.

## Conclusion

The pharyngeal swallowing function might be preserved even in a state of akinetic mutism in patients with MM2C-type sCJD. The pathophysiology of dysphagia is considered to exhibit a pseudobulbar palsy pattern.

## References

[1] Iwasaki Y. Creutzfeldt-Jakob disease. Neuropathology. 2017;37:174–188. 28028861 10.1111/neup.12355

[2] Parchi P, Giese A, Capellari S, et al. Classification of sporadic Creutzfeldt-Jakob disease based on molecular and phenotypic analysis of 300 subjects. Ann Neurol. 1999;46:224–233. 10443888

[3] Hayashi Y, Iwasaki Y, Waza M, et al. Clinicopathological findings of an MM2-corticyal-type sporadic Creutzfeldt-Jakob disease patient with cortical blindness during a course of glaucoma and age-related macular degeneration. Prion. 2019;13:124–131. 31219399 10.1080/19336896.2019.1631680 PMC6629179

[4] Biadri S, Capellari S, Ladogana A, et al. Revisiting the Heidenhain variant of Creutzfeldt-Jakob disease: evidence for prion type variability influencing clinical course and laboratory findings. J Alzheimers Dis. 2016;50:465–476. 26682685 10.3233/JAD-150668 PMC4927903

[5] Hayashi Y, Yamada M, Kimura A, et al. Clinical findings of a probable case of MM2-cortical-type sporadic Creutzfeldt-Jakob disease with antibodies to anti-N-terminus of α-enolase. Prion. 2017;11:454–464. 28967811 10.1080/19336896.2017.1377876 PMC5786357

[6] Hayashi Y, Iwasaki Y, Waza M, et al. Clinicopathological findings of a long-term survivor of V180I genetic Creutzfeldt-Jakob disease. Prion. 2020;14:109–117. 32178563 10.1080/19336896.2020.1739603 PMC7153845

[7] Iwasaki Y, Akagi A, Mimuro M, et al. Factors influencing the survival period in Japanese patients with sporadic Creutzfeldt-Jakob disease. J Neurol Sci. 2015;357:63–68. 26143527 10.1016/j.jns.2015.06.065

[8] Hamaguchi T, Sanjo N, Ae R, et al. MM2-type sporadic Creutzfeldt-Jakob disease: new diagnostic criteria for MM2-cortical type. J Neurol Neurosurg Psychiatry. 2020;91:1158–1165. 32839349 10.1136/jnnp-2020-323231

[9] Master CL, Harris JO, Gajudesk DC, et al. Creutzfeldt-Jakob disease: patterns of worldwide occurrence and the significance of familial and sporadic clustering. Ann Neurol. 1979;5:177–188. 371520 10.1002/ana.410050212

[10] Atarashi R, Satoh K, Sano K, et al. Ultrasensitive human prion detection in cerebrospinal fluid by real-time quaking-induced conversion. Nat Med. 2011;17:165–178. 10.1038/nm.2294 21278748

[11] Yoneda M, Fujii A, Ito A, et al. High prevalence of serum autoantibodies against the amino terminal of alpha-enolase in Hashimoto’s encephalitis. J Neuroimmunol. 2007;185:195–200. 17335908 10.1016/j.jneuroim.2007.01.018

[12] Kunieda K, Ohno T, Fujishima I, et al. Reliability and validity of a tool to measure the severity of dysphagia: the food intake LEVEL scale. J Pain Symptom Manage. 2013;46:201–206. 23159683 10.1016/j.jpainsymman.2012.07.020

[13] Miyaji H, Umezaki T, Adachi K, et al. Videofluoroscopic assessment of pharyngeal stage delay reflects pathophysiology after brain infarction. Laryngoscope. 2012;122:2793–2799. 22965906 10.1002/lary.23588

[14] Hayashi Y, Yoshikura N, Takekoshi A, et al. Preserved regional cerebral blood flow in the occipital cortices, brainstem, and cerebellum of patients with V180I-129M genetic Creutzfeldt-Jakob disease in serial SPECT studies. J Neurol Sci. 2016;370:145–151. 27772745 10.1016/j.jns.2016.09.043

[15] Lang IM. Brain stem control of the phases of swallowing. Dysphagia. 2009;24:333–348. 19399555 10.1007/s00455-009-9211-6

[16] Kunieda K, Hayashi Y, Yamada M, et al. Serial evaluation of swallowing function in a long-term survivor of V180I genetic Creutzfeldt-Jakob disease. Prion. 2020;14:180–184. 32627665 10.1080/19336896.2020.1787090 PMC7518740

